# Investigating the Effectiveness of Buccal Flap for Velopharyngeal Insufficiency: A Systematic Review Article

**DOI:** 10.3390/jcm14082593

**Published:** 2025-04-10

**Authors:** Amr Youssef Arkoubi

**Affiliations:** Department on Anesthesia and Surgery, Faculty of Medicine, Imam Mohammad Ibn Saud Islamic University (IMSIU), Riyadh 11564, Saudi Arabia; ayarkoubi@imamu.edu.sa

**Keywords:** buccal flap, hypernasality, speech intelligibility, speech outcomes, velopharyngeal insufficiency

## Abstract

**Background**: Velopharyngeal insufficiency (VPI) is a failure of the sphincter mechanism, causing speech patterns like hypernasality and decreased intelligibility. Causes include structural, neurologic, and mechanical issues. Treatment options include non-surgical and surgical interventions, but complications can arise. A new approach using the buccal flap (BF) has been suggested for palatal length augmentation. This systematic review assessed speech outcomes after BF palatal lengthening. **Methods**: A thorough investigation was conducted by methodically reviewing numerous databases, including PubMed, Scopus, Web of Science, and Embase, until December 2024. The goal of our analysis was to find studies that assess the short- and long-term efficacy of BF on speech outcomes on patients with VPI. We used the NIH Quality Assessment Tool to assess the quality of the evidence, ensuring the dependability of the results reached during these investigations. **Results**: This systematic review identified 23 studies (total sample size of 995) that assessed the speech outcomes of BF on VPI. The BF significantly improves speech outcomes in patients with VPI. Hypernasality improved significantly post-surgery, with outcomes measured using different scales and methods, including both subjective and objective tools. Benefits were observed within three months postoperatively, with sustained benefits up to 15 months in several studies. Speech intelligibility also improved notably, with mean differences up to 1.09 (*p* < 0.001). Reductions in audible nasal air emissions were observed, though some variability was noted across studies. Secondary outcomes, including better velopharyngeal closure and decreased facial grimacing, further highlight its efficacy. However, inconsistent findings for nasal turbulence and limited long-term data suggest that benefits may plateau over time. These findings support the BF as an effective intervention, though further research is needed on extended outcomes. **Conclusions**: BF is an effective surgical intervention for VPI, significantly improving hypernasality, speech intelligibility, and audible nasal air emissions. While benefits are evident across diverse populations, long-term outcomes and secondary features, such as nasal turbulence, show variability, emphasizing the need for individualized approaches and continued follow-up. This technique offers a reliable option for functional and speech rehabilitation, though further research is needed to optimize its long-term efficacy and broader outcomes.

## 1. Introduction

The velopharyngeal sphincter is composed of the soft palate, lateral pharyngeal walls, and posterior pharyngeal wall. It separates the nasal cavity from the oral cavity during speech [1]. The levator veli palatini, tensor veli palatini, musclus uvulae, palatoglossus, palatopharyngeus, salpingopharyngeus, and superior pharyngeal constrictor muscles have critical roles in the movements of this sphincter [2]. These muscles manipulate air pressure and regulate sound generation from the larynx into intelligible speech by controlling the shape and size of the sphincter and directing the airflow between the oral and nasal resonating cavities [3].

Velopharyngeal insufficiency (VPI) is defined as failure of the sphincter mechanism, which leads to the inability of the nasopharynx to separate sounds between the nasal and oral cavities. This leads to altered speech patterns such as hypernasality, increased nasal emission, and weak consonant production. Speech alteration over time can lead to decreased intelligibility [4]. There are several causes of VPI, including structural, neurologic, and mechanical causes, such as enlarged tonsils [5]. However, in general, VPI is divided into primary and secondary. Primary VPI occurs in patients with cleft palate due to insufficient tissue or displacement of the velopharyngeal structures [6,7,8]. Secondary VPI occurs after primary cleft palate class I or II repair. It remains a surgical challenge in cleft palate care [9,10]. There are various modalities to assess and treat VPI. Multiple speech assessment tools are used to determine the degree of velopharyngeal insufficiency [11,12,13].

As for treatment, there are multiple non-surgical and surgical approaches to treat VPI. Nonsurgical treatment is generally less complex and includes intraoral obturators and speech therapy [14]. As for surgical techniques, there are multiple surgical interventions such as superiorly based pharyngeal flap, sphincter pharyngoplasty, double-opposing Z-palatoplasty, palatal muscle retro positioning, and posterior pharyngeal wall augmentation [9,10,15,16,17,18,19]. However, these approaches can be complicated by obstructive sleep apnea, snoring, mouth breathing, and hypo nasality [20].

A new approach using the buccal flap (BF) has been suggested. The BF has multiple uses in cranio-facial surgery. Buccal flaps have been used for the closure of a palatal fistula and primary and secondary cleft palate repair. It has several advantages, such as minimal donor site morbidity, and it is also harvested easily. Due to the proven benefits, the BF serves as a way of palatal length augmentation [14]. Although BF palatal lengthening surgery has been proven to be safe and efficient as a treatment option for VPI, this technique is still under-utilized as a first treatment option. We performed this systematic review to address the advantages, limitations and complications of BF surgery. Moreover, we aim to assess the speech outcome results measured with standardized assessment tools after BF palatal lengthening.

## 2. Materials and Methods

### 2.1. Protocol and Registration

This systematic review was conducted in adherence to the Preferred Reporting Items for Systematic reviews and Meta-Analyses (PRISMA) guidelines [21] and detailed instructions of Cochrane Handbook for Systematic reviews of Interventions [22].

### 2.2. Eligibility Criteria

#### 2.2.1. Inclusion Criteria

We included prospective and retrospective studies of different study designs in our review if they satisfied the following criteria:

1. Population: Patients with secondary cleft palate repair characterized by VPI and treated using buccal flaps.
2. Intervention: Buccal flap surgery alone or combined with other interventions for palatal lengthening
3. Outcome: We focused on the evaluation of speech outcomes before and after buccal flap surgery.
  i. Primary outcomes: Degree of hypernasality, speech intelligibility and audible nasal air emissions.
  ii. Secondary outcomes: Other speech outcomes, nasopharyngoscopy scores, passive cleft speech characteristics, nasal turbulence, and facial grimacing, in addition to reported complications.

#### 2.2.2. Exclusion Criteria

Reviews, letters to editors, abstracts, opinions, and studies conducted on animals were excluded from our systematic review. Studies that used interventions other than buccal flap surgery, studies focused on the description of surgical technique, studies that did not evaluate speech outcomes, and studies that were not published in English were also excluded.

### 2.3. Information Sources and Search Strategy

A literature search was performed of four databases (PubMed, Cochrane Central Register of Controlled Trials (CENTRAL), web of science, and Scopus) for published articles until December 2024 using search strategy (velopharyngeal dysfunction) or (velopharyngeal insufficiency) OR VPI) and ((buccal flap) or (buccinator flap) or (mucosal buccal flap) or (palatal lengthening)) with minor adjustments according to each database. Our search considered research articles published in the English language only. 

### 2.4. Study Selection and Data Extraction

Search results were exported into Endnote [23] to remove duplicate records. After that, Rayyan software [24] was utilized independently by two authors to independently review titles and abstracts to assess their eligibility for the study. Any disagreement between the two reviewers was solved by discussion. The full-text screening was conducted on the eligible articles which met the inclusion criteria. References of included studies were retrieved to avoid omitting potential additional studies. The data of final records include study ID, location, year of publication, target population, sample size, follow-up duration, details of surgery, and main findings. The assessed outcomes were extracted manually from the articles into a Google sheet and qualitatively synthesized by two independent reviewers.

### 2.5. Assessment of Risk of Bias

We used the National Institutes of Health (NIH) Quality Assessment Tool [25] for Pre-Post studies with no control group to assess the risk of bias in each of the included studies. The NIH tool includes twelve different domains: the clarity of the study objective, eligibility criteria, generalizability, participant sampling, number of participants, the consistency of the intervention without deviations, assessment protocol, blinding of evaluators, attrition, statistical analysis, outcome measurement, and confounding variables. Two authors conducted meticulous assessment for each domain independently and any conflicting decisions were thoroughly discussed.

## 3. Results

### 3.1. Study Selection

**Figure 1:**
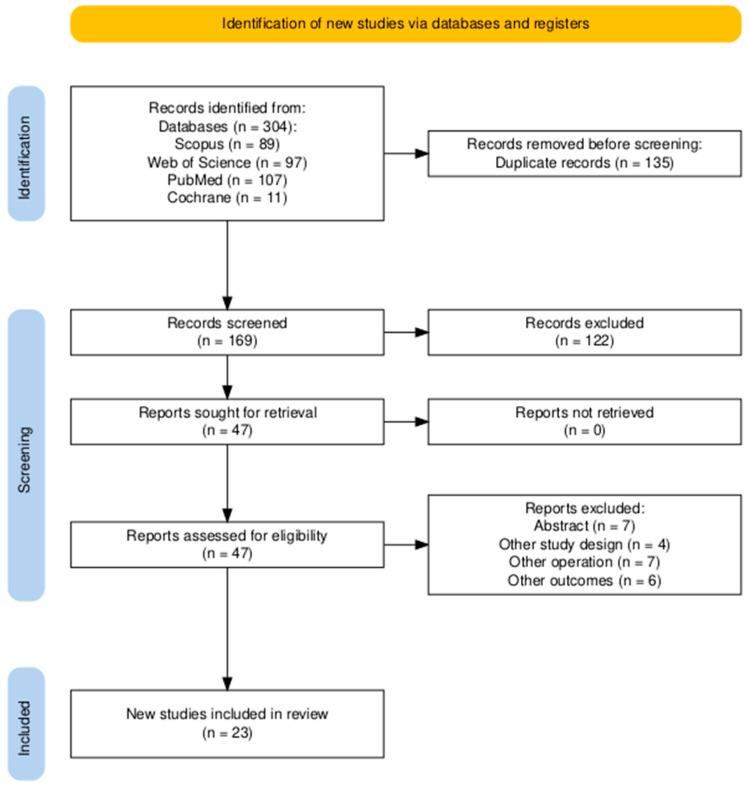
PRISMA flow diagram of searching and screening processes [21].

A comprehensive literature search across the PubMed, Scopus, Cochrane, and Web of Science databases yielded 304 records. Endnote was employed, resulting in the exclusion of 135 duplicate records. Of the remaining 169 records, screening of titles and abstracts resulted in the exclusion of 122 records and the identification of 47 records. Forty-seven records underwent a comprehensive full-text evaluation and were assessed against eligibility criteria, resulting in the exclusion of twenty-four records for various reasons. Ultimately, 23 studies (Aboulhassan 2024 [26], Adeyemo 2019 [27], Ahl 2015 [28], Anstadt 2022 [29], Askar 2024 [30], Celik 2017 [31], Chauhan 2020 [11], Denadai 2018 [32], Denadai 2017 [33], Elsherbiny 2020 [34], Hens 2013 [35], Hill 2004 [36], Hoghoughi 2024 [37], Kimia 2024 [38], Lignieres 2023 [39], Logjes 2016 [40], Mann 2011 [14], Monte 2024 [41], Napoli 2021 [42], Park 2022 [43], Robertson 2008 [44], Sitzman 2024 [45] and Smarius 2021 [46]) were chosen and incorporated into the systematic review. The PRISMA flow diagram representing the selection process is illustrated in Figure 1. 

### 3.2. Characteristics of Studies

The included studies in this systematic review encompass diverse methodologies and settings, reflecting global efforts to address persistent velopharyngeal insufficiency (VPI) following cleft palate repair. The majority are retrospective cohort studies, with a few prospective cohorts and case series included. Sample sizes ranged from 8 to 106 participants, with mean ages between 4.4 and 20.8 years. Surgical interventions varied, including techniques such as Furlow palatoplasty, BF procedures, and pharyngoplasty, tailored to the specific needs of each patient population. Postoperative evaluation times ranged from 1 to 60 months, capturing both short- and long-term outcomes. Most studies reported significant improvements in speech outcomes, including reductions in hypernasality, improved intelligibility, and enhanced velopharyngeal closure. A few studies highlighted surgical innovations, such as the use of buccal fat pads and double-opposing flaps, demonstrating promising results with reduced complications. Collectively, these studies emphasize the importance of individualized surgical approaches to optimize functional and speech outcomes in patients with VPI. A summary of the included studies is provided in Table 1.

### 3.3. Risk of Bias and Quality Assessment

Quality assessment of included studies was conducted using the NIH tool. The evaluation revealed that eight studies (Chauhan 2020, Mann 2011, Adeyemo 2019, Anstadt 2022, Çelik 2017, Napoli 2021, Robertson 2008 and Smarius 2021) [11,14,27,29,31,42,44,46] were judged as having fair quality primarily due to sample size and unblinding of outcome assessor. Moreover, the remaining 14 studies were considered good (Table 2).

### 3.4. Primary Outcomes

The BF has been well investigated with regard to speech-related outcomes, with much focus on hypernasality, speech intelligibility, and audible nasal air emissions. Other outcomes, including nasopharyngoscopy ratings, nasal turbulence, passive cleft speech characteristics, and facial grimacing, offer a broader view of the functional effects this surgical procedure produces. In our analysis, we only included numbers from comparable scoring systems. For studies that used different scales to assess hypernasality, speech intelligibility, and other outcomes, we qualitatively described the improvements (e.g., ‘moderate improvement’ or ‘marked improvement’) to account for the differences in measurement systems. Detailed information about the different scoring systems used across studies is provided in the Supplementary Material. The evidence clearly demonstrates benefits but to varying degrees as a result of follow-up times and patient demographics. Detailed findings of the speech outcomes of each study are presented in Table 3.

#### 3.4.1. Hypernasality

Hypernasality, the primary concern in velopharyngeal dysfunction, was significantly improved after the BF surgery. The hypernasality scale ranged from 0 to 3 (0 = no hypernasality, 1 = mild hypernasality, 2 = moderate hypernasality, 3 = severe hypernasality). Most of the studies reported significant reductions in hypernasality, with a mean difference ranging from 1.08 to 2.5 and *p*-values consistently below 0.001. Improvements were seen as early as three months post-surgery, as illustrated by studies like Aboulhassan (2024) and Denadai (2018).

Sustained benefits were reported up to 15 months in studies like Denadai (2017) and Hens (2013). Additionally, Park (2022) reported a remarkable reduction in hypernasality (mean difference of 2.5, *p* < 0.0001), highlighting the technique’s efficacy.

The degree of improvement varied between patients with syndromic and non-syndromic conditions. Logjes (2016) showed that the syndromic patients had greater reductions (mean difference 1.7, *p* < 0.0001) compared to the non-syndromic patients (mean difference 1.2, *p* < 0.0001), suggesting that patient-related variables could influence outcomes. By contrast, a study by Askar (2024) revealed no significant variation in hypernasality during long-term follow-ups (60 months), which calls for more studies regarding long-term efficacy.

#### 3.4.2. Speech Intelligibility

Results regarding the outcomes of speech intelligibility highlighted the effectiveness of the BF. The speech intelligibility scale ranged from 0 to 3 (0 = within normal limits, 1 = mild speech intelligibility, 2 = moderate speech intelligibility, 3 = severe speech intelligibility). Many studies demonstrated significant improvement in intelligibility scores within a few months after surgical intervention. For example, Aboulhassan (2024) found a mean difference of 1.09 (*p* < 0.001) at three months, whereas Chauhan (2020) found improvements of 0.95 (*p* = 0.001) at six months.

This benefit was found to be more significant for syndromic patients by Logjes (2016), where the mean difference was 1.3 compared with 0.9 in non-syndromic patients. This would hint at the fact that not only does this procedure generally enhance resonance but also speech clarity and articulation.

Some studies with longer follow-up, such as Smarius (2021), showed the benefits to be maintained with a mean difference of 0.8 (*p* < 0.001). However, another study, that of Askar (2024), did not detect any clinically significant changes at 60 months in speech intelligibility, indicating that, in some cases, the gains were substantial at the beginning but started to plateau or deteriorate over time.

#### 3.4.3. Audible Nasal Air Emissions

Audible nasal air emissions, commonly seen as a consequence of velopharyngeal dysfunction, also improved significantly following intervention. The audible nasal emission score ranges from 0 to 3 (0, no; 1, mild “inconsistent, visible”; 2, moderate “consistent, visible”; 3, severe “audible or turbulence”). Denadai (2017) reported consistent decreases in all time frames, with the mean differences reaching 2.0 at 15 months (*p* < 0.001). Similarly, Elsherbiny (2020) recorded significant improvement at six months, with a mean difference of 0.92 (*p* = 0.015). Notably, Hens 2013 and Sitzman 2024 published variable results with minimal to non-significant reductions in nasal emissions. These controversial findings may arise from disparate surgical techniques, assessment procedures, or patient populations.

### 3.5. Secondary Outcomes

#### 3.5.1. Secondary Outcomes and General Functional Implications

The secondary outcomes provided additional insight into the functional improvements afforded by the BF. Nasopharyngoscopy examinations by Aboulhassan (2024) showed great progress with a mean difference of 1.04 (*p* < 0.001), indicating good velopharyngeal closure. Additionally, passive cleft speech features, as outlined by Ahl (2015), showed significant improvements (*p* < 0.001), highlighting how important the procedure is for the correction of structural deficits. Facial grimacing, as a compensatory response to velopharyngeal insufficiency, showed a significant decrease in studies like Elsherbiny (2020), which reported a mean difference of 0.5 (*p* = 0.01). Conversely, results for nasal turbulence were inconsistent, with a number of studies showing no significant changes (e.g., Hens 2013, *p* > 0.05). Such inconsistency suggests that while the BF is effective in addressing primary speech outcomes, some secondary features may require supplementary therapeutic interventions.

#### 3.5.2. Complications

**Furlow Palatoplasty with Buccal Myomucosal Flap:** Aboulhassan 2024 described the complications of buccal fat herniation and wound dehiscence after secondary Furlow palatoplasty with buccal myomucosal flap. Elrouby 2024 also pointed out concerns with soft palate work in relation to speech and middle ear function due to muscle imbalance that may contribute to soft palate dysfunction. Also, the technique was determined to be more difficult with wide clefts because of the closure’s tension problem.

**Double Opposing Buccal Flap:** Chauhan 2020 pointed out the difficulties of mastication, traumatic bite of the flap, marginal necrosis, palatal fistula, tubing of the flap, and dimpling of the cheek as additional complications. Furthermore, this approach necessitates a second operation for flap division, which extends convalescence. Mann 2011 described distal flap necrosis in two cases but emphasized the effectiveness of the technique for enhancement of speech and prevention of obstructive sleep apnea (OSA). Monte 2024 noted the dehiscence of the wounds, limitation of movement of the mouth, partial necrosis of the tissues, and formation of fistulas in some patients.

**Bilateral Buccinator Myomucosal Flaps:** Denadai 2017 and Denadai 2018 did not notice any complications in the application of bilateral buccinator myomucosal flaps. On the other hand, Denadai 2018 claimed to have 29.8% complications, which included some chewing damage and blood supply, without any comparative study on the effectiveness of the various other modifications made to the technique. In the same way, Robertson 2008 noted that the technique was useful in secondary repairs, but two patients still had mild velopharyngeal incompetence and hyperresonance.

**Buccal Fat Pad Flap:** Adeyemo 2019, Ahl 2015 and Anstadt 2022 agree that procedures involving buccal fat pad flap did not cause any particular complications. They see the procedures as safe and efficacious. On the contrary, Kotlarek 2022 stated that the capsule of buccal fat pad was at risk of being lacerated, in which case it may necrose and perforate it if not managed properly.

**Posterior Pharyngeal Flap and Sphincter Pharyngoplasty:** Kimia 2024 reported that SPP was accompanied by increased complication rates such as hyponasality and VP port tightness. On the contrary, PL was associated with less airway complications and appeared to have better efficacy.

**Tissue Augmenting Palatoplasty Using Buccal Myomucosal Flaps:** In comparison with the Furlow Z-plasty, Sitzman 2024 noted that BMMF had a lower correction rate for hypernasality (56% versus 80%). Smarius 2021 noted complications like fistulas, bleeding, infections, and trouble with delayed wound healing and wound dehiscence, particularly in attempts at combining Furlow and buccal flap techniques.

**Palatal Lengthening with Buccal Myomucosal Flaps:** Ulma 2020 and Napoli 2021 showed that palatal lengthening with buccal myomucosal flaps had no real complications and decreased hypernasality with no aggravating of obstructive sleep symptoms. However, Hoghoughi 2024 did observe some cases of partial flap loss with incomplete closure at the donor site, which were treated by secondary intention and no fistula

**Table S2 in Supplementary Materials** shows details of reported complications from the included studies.

### 3.6. Buccal Flap: Palatal Repair vs. Other Causes

The outcome from using the BF technique differed greatly depending on the distinguishing reason for VPI. With reorganized cleft palate, VPI studies showed consistent improvement in hypernasality, speech, and air emission metrics on nasal function psychometrics. For example, in Aboulhassan (2024)’s study, postoperative hypernasality (1.4 ± 0.6) reduced to 0.3 ± 0.6 within three months, giving a difference of 1.08 ± 0.28 (*p* < 0.001) with a mean reduction of 1.08 ± 0.28 for hypernasality post-operation. Ahl (2015) noted that the number of patients with hypernasality improved from 68.5% to 24.1% postoperatively, significantly improving speech sophistication. Also, regarding nasal emission, Adeyemo (2019) and Logjes (2016) were able to sharply reduce air emission while improving speech outcomes significantly. For example, Adeyemo reported an improvement in hypernasality and speech 1 month after surgery.

On the contrary, for other causes of VPI, the results were much less consistent. For instance, Askar (2024) noted no significant improvement in hypernasality at 60 months (*p* = 0.718) and speech was also unremarkable (*p* = 0.887). Denadai (2018) reported a mean increase in hypernasality at 3 months (1.1 ± 0.63, *p* < 0.001) but no increase in hypernasality was seen in the long run.

## 4. Discussion

A cleft palate is a serious deformity that can lead to problems with speech, hearing, facial growth, eating, and psychosocial development. It is recommended that palatoplasty be performed between the ages of 6 and 12 months. There are different techniques of palatoplasty. Cleft palate repair techniques include Veau–Wardill–Kilner V-Y, von Langenbeck, two-flap, alveolar extension palatoplasty, vomer flap, raw area free palatoplasty, etc. The soft palate techniques include intravelar veloplasty, double-opposing Z-plasty, radical muscle dissection, and primary pharyngeal flap [47]. The goal of repair is to close the palate and allow good speech articulation and satisfactory midface development. The success of a palate repair is determined mainly by velopatal function and speech. Failure of repair results in fistula formation and/or velopharyngeal incompetence, and secondary surgery is indicated in this situation [44].

BF is commonly used in cleft palate repair to provide additional lining when the nasal mucosa is inadequate [48]. It has several advantages, as it is flexible, versatile, and provides mucosal, as opposed to skin, cover [49].

### 4.1. Main Findings

Evidence from this systematic review supports the BF as an effective intervention to improve speech outcomes for speakers with velopharyngeal insufficiency. Reductions in hypernasality, improvements in speech intelligibility, and decreases in perceptible nasal air emissions were consistently found across studies, with benefits realized shortly after surgery and often maintained over the longer term. These findings highlight the BF as a valuable surgical intervention for the correction of severe deficits associated with VPI.

The greatest concern in VPI is hypernasality, which improved to a great extent after BF surgery. Outcome varied based on patient-related factors such as syndromic vs. non-syndromic status, with syndromic patients generally showing better improvement and possibly indicating that baseline anatomy or function might affect surgical response. In the long term, some data did show, however, that the benefit of the correction of hypernasality may plateau, and ongoing durability needs to be monitored.

The intelligibility of speech showed an important improvement, suggesting that the BF can positively affect both clarity and articulation. More remarkable improvements were especially found in the first months after surgical intervention, with several papers highlighting the significant benefit derived by patients. In many ways, similar to hypernasality, syndromic patients appeared to achieve greater improvements compared with the non-syndromic group. However, some evidence suggested that these may degrade over extended follow-up intervals, and therefore, a clear need for long-term assessment and potentially additional treatment does exist.

Audible nasal air emissions, another important symptom of VPI, showed, in most studies, consistent improvement. The majority of findings supported the efficacy of BF in reducing this issue; however, variability across some reports suggested the influence of factors such as surgical technique and patient selection.

The secondary outcomes provided more detailed knowledge of the comprehensive functional benefits related to BF. Improvements in velopharyngeal closure were typically reported, along with improvements in structural speech features and reduction in compensatory behaviors such as facial grimacing. The results regarding nasal turbulence, on the other hand, were somewhat variable, suggesting that this particular aspect may require either additional interventions or alternative procedures. 

It is critical to discuss the challenges of the different surgical methods used for palatal repair and for treatment of velopharyngeal insufficiency (VPI). While many methods, like buccal fat pad flaps and bilateral buccinator myomucosal flaps, have good results and few complications, other methods, such as the double-opposing buccal flap and the secondary Furlow palatoplasty, have greater complication risks, which include flap necrosis, wound dehiscence, and palatal fistulas. Other procedures, such as posterior pharyngeal flaps and sphincter pharyngoplasty, may increase complication risks of hyponasality and VP port tightness, while palatal lengthening with buccal myomucosal flaps has been shown to decrease hypernasality without worsening obstructive sleep apnea. Therefore, a thorough assessment of individual patient factors is critical in determining the most appropriate surgical technique that carries the least risks and most favorable results.

The variability in outcomes, especially for secondary features and long-term effectiveness, points out that surgical interventions should be tailor-made according to the specific characteristics of the patients. Finally, whereas BF is strikingly effective for primary speech-related deficits, its use for more general functional impairments deserves further exploration. Standardization of outcomes should be one of the priorities in the future, along with researching long-term effectiveness.

The effectiveness of BP varies based on the etiology of VPI. For cases related to cleft palate, marked improvements were noted, especially with hypernasality and speech clarity. However, results were more mixed in other studies that reported little value in long-term outcomes. These findings indicate that the BF technique has merit in addressing cleft VPI, but its merit in non-cleft cases requires much more study.

### 4.2. Clinical Implications

The BF is a valuable surgical option for managing velopharyngeal insufficiency, offering significant improvements in hypernasality, speech intelligibility, and audible nasal air emissions. However, there is insufficient evidence to show that the BF technique provides better outcomes than other techniques for cleft palate repair, and direct comparison studies between these techniques have not been performed. Its effectiveness across diverse patient populations highlights its versatility, though outcomes may vary based on syndromic status and follow-up duration. The procedure also addresses compensatory mechanisms, such as facial grimacing, and enhances velopharyngeal closure. However, variability in long-term efficacy and secondary outcomes, like nasal turbulence, underscores the need for individualized surgical planning and potential adjunctive therapies. Standardized assessments and targeted interventions can further optimize its benefits, ensuring comprehensive functional and speech rehabilitation.

### 4.3. Previous Research

Lentskevich [50] conducted a similar systematic review evaluating the efficacy of the BF in managing velopharyngeal insufficiency, focusing on its impact on speech outcomes. His findings indicated identical conclusions, highlighting the superior effectiveness of the buccal flap in improving hypernasality, speech intelligibility, and audible nasal air emissions. However, Lentskevich’s study faced several limitations, including reliance on results from conference abstracts and the exclusion of numerous relevant studies. Additionally, his review was based on only 13 studies, while our review includes 23 studies, offering a more comprehensive and robust analysis. These differences strengthen the validity of our findings by incorporating a broader and more diverse dataset.

### 4.4. Strengths and Limitations

This systematic review is an overview of the effectiveness of the BF in different groups of patients, with important information about the impact on crucial speech parameters such as hypernasality, speech intelligibility, and detectable nasal air emissions. This review enables a much broader understanding of immediate and long-term effects by including studies with different methodological approaches and follow-up periods. Furthermore, secondary outcomes, such as velopharyngeal closure and compensatory mechanisms, enrich the functional evaluation and provide a global view of the benefits associated with the procedure.

This study has some limitations, such as heterogeneity among the included studies, especially regarding patient demographics, surgical methodologies, and outcome measurements, not to mention follow-up periods and reporting or lack thereof of secondary outcomes, such as nasal turbulence, giving reasons to limit the generalizability of the findings to a great extent. Most of the studies had the limitation of a retrospective nature, so the overall evidence level is low, meaning that it points out the need for more standardized studies that are prospective in order to establish these findings more reliably.

## 5. Conclusions

This systematic review illustrates the effectiveness of the BF in improving speech outcomes for patients with VPI. The procedure consistently reduces hypernasality, improves speech intelligibility, and decreases audible nasal air emissions. Additional advantages, such as enhanced velopharyngeal closure and diminished compensatory strategies, further reinforce its functional effectiveness. Nevertheless, the different results observed in specific secondary outcomes, including nasal turbulence, necessitate additional investigation. Finally, further RCTs are required to evaluate surgery efficacy and obtain higher levels of evidence.

## Figures and Tables

**Table 1:** A more detailed summary of the included studies.

Author, Year	Country	Study Type	Sample Size	Mean Age (yrs)	Indications	Surgery Performed	Average Time to Post-Oprative Evaluation (mos)	Significant Findings
Aboulhassan 2024 [26]	Egypt	Retrospective cohort study	23	6	Treatment of VPI in patients with a cleft palate who were treated with TFP in their primary palate repair.	Secondary Furlow palatoplasty with FPBF	3	Significant improvement was observed regarding the degree of hypernasality and speech intelligibility
Adeyemo 2019 [27]	Nigeria	Case series	8	6.1	secondary palatal cleft repair, those with wide palatal clefts or patients whose primary palatal cleft repair was complicated intraoperatively by inadvertent tearing of the nasal mucosa	Surgery using BFP	1	It is recommended that cleft surgeons add the technique of buccal fat pad application to their armamentarium in difficult cases, especially in wide palatal cleft repair, secondary palatal cleft repair and in cases of inadvertent tearing of nasal mucosa during primary cleft palate repair.
Ahl 2016 [28]	UK	Retrospective cohort	103	5.5	correction of VPD	Unilateral BMF, some with Z-plasty; Bilateral BMF, some with Z-plasty.	6	Significant reduction in VPD from 68.5% patients preoperatively to 24.1% patients postoperatively
Anstadt 2022 [29]	USA	Retrospective cohort	20	9.7	Persistent VPI following primary palatoplasty	Augmentation using BMF	8.9	In patients with VPI following primary palatoplasty, revision palatoplasty with tissue augmentation using BMF offers an alternative to pharyngoplasty. This approach preserves dynamic velopharyngeal function and improves speech outcomes.
Askar 2024 [30]	Egypt	Retrospective cohort	79	13.03	Persistent VPI after primary cleft palate repair.	Furlow palatoplasty or posteriorly based buccal myo-mucosal flaps	60	Different techniques of primary or secondary repair of the cleft palate could jeopardize the nerve supply of the palate, leaving behind a deceiving intact but weak poor-functioning palate. Although the postoperative weak palate is multifactorial, all efforts should be made to preserve the innervation of the palate
Celik 2017 [31]	Turkey	Retrospective cohort	17	-	persistent VPI after primary cleft palate repair	Unilateral BMF, some with IVV; Bilateral BMFs, some with IVV	6	All 17 patients who underwent SPE showed improvement in speech, from very poor to poor speech and from normal to good speech.
Chauhan 2020 [11]	India	Prospective cohort	50	14	persistent VPI after primary cleft palate repair	Bilateral BMFs	7	Significant speech improvement. None of the patients showed hyponasal speech postoperatively
Denadai 2017 [32]	Brazil	Prospective cohort	37	20.8	VPI with moderate or large velopharyngeal gaps; prior VPI surgery	Bilateral BMFs	12	postoperative period showed that hypernasality was significantly lower than the hypernasality of the preoperative and recent postoperative periods
Denadai 2018 [33]	Brazil	Prospective cohort	53	19.3	VPI with moderate or large velopharyngeal gaps; prior VPI surgery	Bilateral BMFs	15	Significant speech improvement. None of the patients showed hyponasal speech postoperatively
Elsherbiny 2020 [34]	Egypt	prospective cohort	30	10.75	Short palate; prior surgery for VPI; anteriorly positioned levator knee; soft palate scarring	Bilateral BMFs with IVV	6	Significant improvements in speech and facial grimace, velar length, closure ratio, velopharyngeal gap, palatal thickness, and mobility
Hens 2013 [35]	Belgium	Retrospective cohort	32	8	short palate; oronasal fistula; soft palate scarring	Bilateral BMFs some with IVV	9.2	Significant decreases in hypernasality ratings and passive cleft characteristics (articulation errors) postoperatively
Hill 2004 [36]	UK	Prospective cohort	16	8.5	short palate; prior surgery for VPI	Bilateral BMFs	12	Ninety-three percent (15 of 16) had a significant improvement in velopharyngeal insufficiency, and 14 patients had no hypernasality postoperatively.
Hoghoughi 2024 [37]	Iran	Cross-sectional clinical study	26	8.5	persistent VPI after primary cleft palate repair	bilateral buccinator flaps coupled with the posterior positioning of the levator veli palatini muscles	6 to 12	12 patients showed a complete resolution of hypernasality, while 9, 3, and 2 patients exhibited mild, moderate, and severe hypernasality.
Kimia 2024 [38]	USA	Retrospective cohort	97	9.62	persistent VPI after primary cleft palate repair	posterior pharyngeal flap (PPF), sphincter pharyngoplasty (SPP), Furlow palate re-repair, and buccal myomucosal flap palate lengthening (PL).	3.93 yrs	Furlow re-repair reduced need for additional VPD operations. Speech outcomes between non-revisional operations are comparable, but increased complications were seen in SPP
Lignieres 2023 [39]	USA	Retrospective cohort	77		persistent VPI after primary cleft palate repair	Buccal flaps with conversion Furlow palatoplasty	3.4 yrs	This study demonstrates that the use of buccal flaps in conversion Furlow palatoplasty could result in lowered risk for postoperative complications as fistulas infection and additional revision surgery despite not significantly alter speech outcomes.
Logjes 2017 [40]	The Netherlands	Retrospective cohort	42	4.9	Persistent VPI after primary cleft palate repair	Unilateral BMF with Z-plasty	14.4	Sufficient speech outcome was achieved in 83% patients with significant improvements in velopharyngeal closure, intelligibility, and resonance
Mann 2011 [14]	USA	Retrospective cohort	27	10.3	VPI with good velar movement; small velopharyngeal gap (<5 mm)	Bilateral BMFs	58	Significant speech improvement. None of the patients showed hyponasal speech postoperatively
Monte 2024 [41]	Brazil	Retrospective cohort	106	-	persistent VPI after primary cleft palate repair	palatal lengthening via double opposing buccinator myomucosal flaps	15	palatal lengthening via double opposing buccinator myomucosal flaps similarly improves speech outcomes.
Napoli 2024 [42]	USA	Prospective cohort	10	9.1	persistent VPI after primary cleft palate repair	Bilateral BMFs	-	Reduction in hypernasality and improvements in speech acceptability and audible nasal emission
Park 2022 [43]	Republic of Korea	Retrospective cohort	32	5.4	persistent VPI after primary cleft palate repair	Furlow double-opposing Z-plasty alone or combined with a BFP	12.7	Most patients who received a BFP showed improvement in hypernasality.
Robertson 2008 [44]	USA	Prospective cohort	22	8.5	Short palate; oronasal fistula; poor speech	Unilateral BMF, some with pharyngoplasties or Z-plasty	1	No fistulas remained. Overall speech quality was significantly improved
Sitzman 2023 [45]	USA	Retrospective cohort	32	5.9 (median)	Persistent VPI after primary cleft palate repair	Secondary Furlow (Furlow) or BMMF	12	Furlow and BMMF procedures increase velar length with favorable speech outcomes. The same degree of improvement for hypernasality was observed across groups,
Smarius 2021 [46]	The Netherlands	Retrospective cohort	56	4.4	primary cleft palate repair for submucous cleft palate	Furlow plasty, intravelar veloplasty, pharyngoplasty, or Furlow combined with buccal flap	14.5	Any child presenting with repeated episodes of otitis media, nasal regurgitation, or speech difficulties should have prompt consideration for SMCP as diagnosis.

Abbreviations: VPI: velopharyngeal insufficiency, TFP: two-flap palatoplasty, FPBF: buccal myomucosal flap, BFP: buccal fat pad, VPD: velopharyngeal dysfunction, BMF: buccal mucosal flap, IVV: intravelar veloplasty, SPE: secondary palatal elongation, CP: cleft palate, LVP: levator veli palatini, VP: velopharyngeal, BMMF: buccal myomucosal flaps, SMCP: submucous cleft palate.

**Table 2:** The risk of bias table using the NIH quality assessment tool.

Study ID	Quality Assessment	Study ID	Quality Assessment
Aboulhassan 2024 [26]	Good	Anstadt 2022 [29]	Fair: small sample size; unclear blinding of outcome assessor and inadequate follow-up period
Hoghoughi 2024 [37]	Good	Smarius 2021 [46]	Fair: small sample size; unclear blinding of outcome assessor and method of speech quality evaluation
Sitzman 2023 [45]	Good	Napoli 2021 [42]	Fair: small sample size and follow-up unknown
Robertson 2008 [44]	Fair: small sample size; Outcome assessors were unblinded	Elsherbiny 2020 [34]	Good
Monte 2024 [41]	Good	Chauhan 2020 [11]	Fair: outcome assessors were unblinded; lack of frequent and consistent follow-up period
Askar 2024 [30]	Good	Adeyemo 2019 [27]	Fair: small sample size and follow-up unknown and unclear blinding
Kimia 2024 [38]	Good	Denadai 2017 [32]	Good
Lignieres 2024 [39]	Good	Denadai 2018 [33]	Good
Çelik 2017 [31]	Fair: small sample size; lack of information on outcome assessor and method of speech quality evaluation	Ahl 2016 [28]	Good
Park 2022 [43]	Good	Mann 2011 [14]	Fair: single outcome assessor was unblinded
Logjes 2017 [40]	Good	Hill 2004 [36]	Good
Hens 2013 [35]	Good		

**Table 3:** The outcome findings of the included studies.

Study and Outcomes Assessed *	Period of Assessment Post-Surgery	Pre-Surgery	Post-Surgery	Mean Difference (MD)	*p*-Value
**Aboulhassan 2024 [26]**					
Hypernasality	3 months	1.4 ± 0.6	0.3 ± 0.6	1.08 ± 0.28	<0.001
Speech Intelligibility	3 months	1.3 ± 0.5	0.3 ± 0.4	1.09 ± 0.27	<0.001
Nasopharyngoscopy scores	3 months	2.8 ± 0.4	3.9 ± 0.3	1.04 ± 0.20	<0.001
**Ahl 2016 [28]**					
Hypernasality	14 ± 9 months			Marked reduction	<0.001
Speech Intelligibility	14 ± 9 months			Significant reduction	<0.05
Passive Cleft Speech Characteristics:	14 ± 9 months			Marked improvement	<0.001
Nasal Turbulence	14 ± 9 months			No significant change	0.13
**Anstadt 2022 [29]**					
Pittsburgh Weighted Speech Score (PWSS)	8.9 months	14.3 ± 4.9	4.2 ± 2.3	10.1± 3.36	<0.001
**Askar 2024 [30]**					
Hypernasality	60 months			No Significant difference	0.718
Speech Intelligibility	60 months			No Significant difference	0.887
Pharyngeal articulation	60 months			No Significant difference	0.622
Audible nasal emission	60 months			No Significant difference	0.442
Facial grimacing	60 months			No Significant difference	0.627
**Chauhan 2020 [11]**					
Hypernasality	6 months	2.1 ± 0.43	0.86 ± 0.47	1.2 ± 0.3	0.001
Speech Intelligibility	6 months	2.02 ± 0.51	1.07 ± 0.47	0.95 ± 0.3	0.001
Denadai 2017 [32]					
Hypernasality	3 months	2.8 ± 0.4	1.7 ± 0.9	1.1 ± 0.63	<0.001
Hypernasality	6 months	2.8 ± 0.4	0.5 ± 0.7	2.3 ± 0.45	<0.001
Denadai 2018 [33]					
Hypernasality	3 months	2.7 ± 0.5	1.5 ± 1	1.2 ± 0.67	<0.001
Hypernasality	6 months	2.7 ± 0.5	1.2 ± 0.9	1.5 ± 0.58	<0.001
Hypernasality	12 months	2.7 ± 0.5	0.5 ± 0.7	2.2 ± 0.42	<0.001
Hypernasality	15 months	2.7 ± 0.5	0.4 ± 0.6	2.3 ± 0.36	<0.001
Audible nasal air emission	3 months	2.2 ± 0.8	0.7 ± 0.8	1.5 ± 0.51	<0.001
Audible nasal air emission	6 months	2.2 ± 0.8	0.6 ± 0.7	1.6 ± 0.48	<0.001
Audible nasal air emission	12 months	2.2 ± 0.8	0.3 ± 0.5	1.9 ± 0.50	<0.001
Audible nasal air emission	15 months	2.2 ± 0.8	0.2 ± 0.4	2.0 ± 0.54	<0.001
**Elsherbiny 2020 [34]**					
Hypernasality	6 months	2.29 ± 0.69	0.96 ± 0.69	1.33 ± 0.44	0.001
Speech Intelligibility	6 months	2.33 ± 0.76	1.08 ± 0.72	1.25 ± 0.47	<0.001
Audible nasal air emission	6 months	2.04 ± 0.95	1.12 ± 0.80	0.92 ± 0.57	0.015
Facial grimacing	6 months	1.79 ± 0.93	1.29 ± 1.08	0.50 ± 0.65	0.01
**Hens 2013 [35]**					
Hypernasality	9.2 months [5.7 months to 17.2 months]	2.4 ± 1.4	0.3 ± 0.8	2.1 ± 0.9	<0.0001
Audible nasal air emission	9.2 months [5.7 months to 17.2 months]	0.7 ± 0.8	0.3 ± 0.6	0.4 ± 0.5	>0.05
Nasal turbulence	9.2 months [5.7 months to 17.2 months]	0.4 ± 0.7	0.8 ± 0.8	0.4 ± 0.48	>0.05
**Lignieres 2024 [39]**					
Hypernasality	NR	2.00 ± 1.29	0.86 ± 1.21	1.14 ± 0.79	<0.001
**Logjes 2017 [40]**					
Hypernasality *(syndromic patients)*	14.4 months	2.1 ± 0.5	0.4 ± 0.5	1.7 ± 0.32	<0.0001
Speech Intelligibility *(syndromic patients)*	14.4 months	3.2 ± 0.8	1.9 ± 0.9	1.3 ± 0.55	<0.0001
Hypernasality *(non-syndromic)*	14.4 months	2 ± 1	0.8 ± 1	1.2 ± 0.63	<0.0001
Speech Intelligibility *(non-syndromic)*	14.4 months	3.5 ± 0.9	2.6 ± 0.9	0.9 ± 0.57	<0.0001
**Mann 2011 [14]**					
Hypernasality	NR	2.15 ± 0.5	0.65 ± 0.5	1.5 ± 0.32	<0.0001
**Park 2022 [43]**					
Hypernasality	NR	4.1 ± 1.0	1.6 ±1.1	2.5 ± 0.67	<0.0001
**Sitzman 2023 [45]**					
Hypernasality	NR	NR	NR	2 ± 1.6	<0.05
Audible nasal air emission	NR	NR	NR	0. 75 ± 1.41	<0.05
**Smarius 2021 [46]**					
Speech Intelligibility	NR	3.5 ± 0.69	2.7 ± 0.92	0.8 ± 0.55	<0.001

* Scores used for speech outcome assessment are described in detail in the Supplementary Materials.

## Data Availability

The datasets analyzed during the current study are available from the corresponding author upon reasonable request.

## Supplementary Materials

The following supporting information can be downloaded at: https://www.mdpi.com/article/10.3390/jcm14082593/s1.
