# Supplementary material for: Investigating the Effectiveness of Buccal Flap for Velopharyngeal Insufficiency: A Systematic Review Article
Source: J Clin Med. 2025 Apr 10;14(8):2593. doi: 10.3390/jcm14082593 (PMC12027865; doi:10.3390/jcm14082593)
Supplement: Supplementary file 1 [file jcm-14-02593-s001.zip › jcm-3486486-supplementary.pdf]

Scores used to measure primary and secondary outcomes.

Aboulhassan 2024

- The hypernasality scale ranged from 0 to 3 (0 = no hypernasality, 1 = mild hypernasality, 2 = moderate hypernasality, 3 = severe hypernasality)
- The speech intelligibility scale ranged from 0 to 3 (0 = within normal limits, 1 = mild speech intelligibility, 2 = moderate speech intelligibility, 3 = severe speech intelligibility)
- Nasopharyngoscopy scores: The four-point scale ranged from 0 to 4 with 0 = No closure, 1 = midway between 0–2, 2 = Incomplete closure (halfway closure), 3 = midway between 2–4, 4 = complete velopharyngeal closure.

Ahl 2015

Speech assessment using the speech tool Cleft Audit Protocol for Speech—Augmented (CAPS-A)

Table 2 CAPS-A Data Categorization[a]

	VPD Severity Score				
Parameter	*Not Present*		*Mild*	*Marked*	
Hypernasality	0	1	2	3	4
Audible nasal emission	0	1		2	
Nasal turbulence	0	1		2	
Passive cleft speech characteristics	0		1	2	

[a] VPD = velopharyngeal dysfunction.

Anstadt 2022

Pittsburgh Weighted Speech Score (PWSS): 0 signifies competence, 1–2 borderline competence, 3–6 borderline incompetence, and 7 or greater represents incompetence or VPI. Scores above 6 are considered to be socially stigmatizing.

Askar 2024

Auditory perceptual assessment (APA):

APA	Hn<br>- mild<br>- moderate<br>-severe<br>PhA<br>- present:<br>- absent:<br>FG<br>- present:<br>- absent:<br>NEm<br>- present:<br>- absent:<br>SI<br>- good<br>- mild impairment<br>- moderate impairment<br>-severe impairment

Hn: Degree of hypernasality; PhA: Pharyngeal articulation; NEm: Audible nasal emission; FG: facial grimacing; SI: Overall Speech intelligibility, good SI means understandable speech while severe impairment means difficult or non-understandable.

Chauhan 2020

Table 1
Severity ratings and corresponding descriptors for hypernasality and intelligibility.

Rating	Severity	Hypernasality	Intelligibility
0	Normal	There is no perceptual evidence of cleft-type speech	Speech is always easy to understand
1	Mild	Nasality is perceived as socially acceptable and the patient/parents are satisfied with the individual's speech resonance	Speech is occasionally hard to understand
2	Moderate	Increased nasality heard on high and low vowels; speech is socially unacceptable	Speech is often hard to understand
3	Severe	Increased nasality heard on vowels and some voiced consonants; speech is socially very unacceptable	Speech is hard to understand most or all of the time

Denadai 2018

- The hypernasality scale ranged from 0 to 3 (0 = no hypernasality, 1 = mild hypernasality, 2 = moderate hypernasality, 3 = severe hypernasality)

Denadai 2017

Parameter (score)	Definition	References
**Velopharyngeal gap size**		
Complete velopharyngeal closure (0)	Complete velopharyngeal closure	
Pinhole (1)	Mucus bubbling	4-6
Small (2)	Velopharyngeal closure > 80% and < 100%	
Moderate (3)	Velopharyngeal closure between 50% and 80%	
Large (4)	Velopharyngeal closure < 50%	
**Hypernasality** *		
None (0)		
Mild (1)		
Moderate (2)		
Severe (3)		
**Audible nasal air emission** *		
None (0)		4,27-30
Mild (1)		
Moderate (2)	–	
Severe (3)		

Elsherbiny 202

Nasality (0, no hypernasality; 1, mild [also includes inconsistency]; 2, moderate; 3, severe), nasal emission (0, no; 1, mild “inconsistent, visible”; 2, moderate “consistent, visible”; 3, severe “audible or turbulence”), weak consonants (0, normal production; 1, weak), intelligibility of speech (0, normal; 1, different from other children’s speech, but not enough to cause comments; 2, different enough to cause comments but possible to understand most speech; 3, only just intelligible to strangers; 4, impossible to understand), grimace (0, no grimace; 1, nasal flare; 2, nostrils and midface; 4, midface and upper face), glottal articulation (0, absent; 1, present), and pharyngealization of fricatives (0, absent; to 1, present).

Hens 2013

CAPS-A: Hypernasality was scored on a 5-point scale, with 0 representing normal; 1, borderline; 2, mild; 3, moderate; and 4, severe. Each of these scalar points had a detailed operational definition. Audible nasal emission and nasal turbulence were each scored on a 3-point scale, with 0 representing absence of symptoms; 1, occasional symptoms; and 2, frequent symptoms. Hyponasality was scored on a 3-point scale, with 0 representing absence of hyponasality; 1, mild hyponasality; and 2, marked hyponasality.

Lignieres 2023

Universal parameters for reporting (UPR) speech outcomes. The UPR uses a four-point scale for hypernasality (0 = within normal limits and 3 = severe hypernasality).

Logjes 2016

- The nasality was graded on a scale ranging from 0 (normal nasality) to 3 (severe hypernasality).
-

Intelligibility score used by parents.

1	Speech is understandable and normal
2	Speech differs from other children. This does not lead to comments and speech is understandable
3	Speech differs from other children. This leads to comments, but speech is understandable
4	Speech is poorly understandable
5	Speech is not understandable

Table 4
Intelligibility score used by speech-language pathologist in the Wilhelmina Children's Hospital.

1	Always understandable for everybody without difficulty
2	Speech-disorder hearable, although understandable
3	Speech-disorder hearable, understandable with some difficulty
4	Speech-disorder hearable, understandable for family with some difficulty, however poorly understandable for strangers despite effort
5	Barely or not understandable for anyone despite effort

Mann 2011

Nasality was graded on a scale ranging from 0 (no hypernasality) to 3 (severe hypernasality).

Park 2022

Hypernasality was stratified into six grades according to severity (normal, grade 0; mild, grade 1; mild-to-moderate, grade 2; moderate, grade 3; moderate-to-severe, grade 4; and severe, grade 5)

Sitzman 2024

- Hypernasality was scored on a 5-point scale, with 0 representing normal; 1, borderline; 2, mild; 3, moderate; and 4, severe.
- Audible nasal emission was scored on a 3-point scale, with 0 representing absence of symptoms; 1, occasional symptoms; and 2, frequent symptoms.

Smarius 2021

Children's Hospital	
1	Always understandable for everybody without difficulty
2	Speech-disorder hearable, although understandable
3	Speech-disorder hearable, understandable with some difficulty
4	Speech-disorder hearable, understandable for family with some difficulty, however poorly understandable for strangers despite effort
5	Barely or not understandable for anyone despite effort

Table S1 shows indications and contraindications for different procedures used to treat Velopharyngeal Insufficiency.

Procedure	Indication	Contraindication
**Surgical procedures**		
**Sphincter Pharyngoplasty**	- Severe or complex VPI, particularly with lateral wall deficiency or poor velar movement.	- Severe nasal airway obstruction. - Significant facial dysmorphology. - Inappropriate for younger children with developing anatomy.
**Pharyngeal Flap Surgery**	Inadequate velopharyngeal closure due to a short or immobile soft palate	Not recommended for complete cleft palates.<br>- Patients with significant airway obstruction
**Palatal Lengthening**	- Short soft palate<br>- Congenital causes submucous cleft palate	- Not effective in elderly due to rigidity of palatal tissue. - Risk of complications, infections or scars
**Furlow Palatoplasty**	- Short or insufficient soft palate. - Used in cleft palate repair or as a revision surgery -Secondary cleft palate repair with small gap (<5 mm to 1 cm)	- Extensive scarring or poor healing capacity especially in immunocompromised patients.
**Injection Augmentation**	- Mild to moderate VPI (Fat injection, Teflon in past, silicone)<br>- Less invasive procedure	- Allergies to injected materials (e.g., Teflon). - High risk of tissue rejection or infection.
**Non-Surgical procedures**		
**Speech Therapy**	- Mild VPI, often related to speech habits<br>- Appropriate for children and post-surgical to improve speech<br>- Poor surgical candidates	- Contraindication is limited<br>- Not suitable when VPI is caused by anatomical issues (e.g., cleft palate).

https://doi.org/10.1016/j.coms.2015.12.004

https://doi.org/10.1016/j.otc.2012.03.005

https://doi.org/10.1097/scs.0000000000009531

https://doi.org/10.1097/moo.0000000000000901

Table S2 showing reported complications and drawbacks

Study ID	Type of Procedure	Complications and Drawbacks
Aboulhassan 2024	Secondary Furlow palatoplasty with buccal myomucosal flap	Buccal fat herniation, buccal wound dehiscence. Lack of comparative analysis with other techniques like pharyngeal flaps.
Adeyemo 2019	Buccal fat pad flap	No specific complications found.
Ahl 2015	Buccinator mucomuscular flap	No specific complications found.
Anstadt 2022	Tissue augmenting palatoplasty using buccal myomucosal flaps	No major complications reported.
Askar 2024	Furlow palatoplasty and posteriorly based myo-mucosal buccal flaps	No specific complications found.
Celik 2017	Secondary palatal elongation (SPE)	No specific complications found.
Chauhan 2020	Double opposing buccal flap	Difficult mastication, traumatic bite of flap, marginal necrosis, palatal fistula formation, tubing of flap, dimple formation at the cheek. Requires second surgical procedure for flap division.
Denadai 2017	Bilateral buccinator myomucosal flaps	No specific complications found.
Denadai 2018	Bilateral buccinator myomucosal flap	29.8% complication rate, flap damage due to chewing, compromised blood flow. No comparative analysis on efficiency of technique modifications.
Elrouby 2024	Furlow Z-palatoplasty with buccal myomucosal flap	Potential issues with soft palate function due to muscle misalignment, leading to compromised speech and middle ear function. Not well-suited for wide clefts due to tension issues.
Elsherbiny 2020	Buccinator flap + radical repositioning of levator muscle	No ischemic complications reported; minor dehiscence in a few cases.
Hens 2013	Buccinator myomucosal flaps for palate lengthening	Some cases of persistent velopharyngeal insufficiency (VPI), require additional interventions.
Hill 2004	Buccinator sandwich pushback	One case of intraoperative vessel injury leading to ischemic flap, one case of postoperative bleeding requiring re-exploration.
Hoghoughi 2024	Modified Approach (Posterior Positioning of Levator Veli Palatini with Intact Nasal Layer and Side-by-Side Bilateral Buccinator Flaps)	3 patients had partial flap loss in one flap; the flaps were repaired with a secondary intention without forming a fistula. In 5 cases, complete closure of the donor sites was not possible; they were repaired with a secondary intention. Also, food-eating restrictions were present in 7 cases following the operation, which were resolved after one month.
Kimia 2024	Posterior pharyngeal flap (PPF), sphincter pharyngoplasty (SPP), palate lengthening (PL)	SPP was associated with increased complication rates, hyponasality, and VP port tightness, while PL had fewer airway complications.
Kotlarek 2022	Pedicled buccal fat pad flap during primary palatoplasty	Risk of necrosis and perforation if capsule of buccal fat pad is lacerated.
Lignieres 2023	Conversion Furlow palatoplasty with buccal flaps	Reduced risk of postoperative fistula compared to traditional Furlow palatoplasty.

Logjes 2016	Unilateral myomucosal buccinator flap	One case of partial flap necrosis requiring revision; potential risk of obstructive sleep apnea (OSA) in cleft patients.
Mann 2011	Double-opposing buccal flap for palatal lengthening	Distal flap necrosis in two cases; generally effective for speech improvement and avoiding OSA.
Monte 2024	Double-opposing buccinator myomucosal flaps	Patient complications from this study (n = 106) included wound dehiscence that healed spontaneously in 3 patients, temporary limitation of mouth opening resolved with pedicle division in 3 additional patients, donor site hematoma in 2 patients, partial necrosis of the distal portion of the flaps in 4 additional patients, 9 patients with a fistula at the Pittsburgh zone III, and 2 patients with a fistula at the Pittsburgh zone IV
Morrison 2024	Buccal Flap	The complication rate was 13.6%: 2 (9.1%) fistulas and 1 (4.5%) superficial wound dehiscence, which was successfully treated with observation. There were no bleeding complications and no episodes of airway obstruction. On patient (4.5%) had a 30-day readmission for an unrelated condition (RSV bronchiolitis) (Table 2). There were no buccal flap losses, no parotid duct injuries, and no facial nerve injuries.
Napoli 2021	Bilateral Buccal Flap Revision Palatoplasty (BBFRP)	No significant complications reported.
Park 2022	Double Opposing Z-Plasty (DOZ) and Buccal Fat Pad (BFP)	No surgical complications such as postoperative bleeding, infection, flap necrosis, flap dehiscence, oronasal fistula, or airway problems were observed in the BFP group. However, one patient in the DOZ group developed an oronasal fistula in the hard palate and soft palate junction 8 months after his/her VPI surgery, which required additional surgery for closure.
Robertson 2008	Buccal Myomucosal Flap in Secondary Cleft Palate Repair	Two patients showed mild velopharyngeal incompetence and continued hyperresonance.
Sitzman 2024	Secondary Furlow Z-Plasty and Buccal Myomucosal Flaps (BMMF)	BMMF had a lower success rate for hypernasality correction compared to Furlow Z-Plasty (80% vs. 56%).
Smarius 2021	Furlow Plasty, Intravelar Veloplasty, Pharyngoplasty, or Furlow Combined with Buccal Flap	Reported complications included fistulas, bleeding, infection, delayed wound healing, and wound dehiscence.
Ulma 2020	Palatal Lengthening with Buccal Myomucosal Flaps	No significant complications reported.
Wu 2020	Double Opposing Z-Plasty (DOZP) for Velopharyngeal Insufficiency (VPI)	Some patients had unsatisfactory outcomes.
